# Anti-Inflammatory and Antioxidant Effects of Dietary Supplementation and Lifestyle Factors

**DOI:** 10.3390/antiox10030371

**Published:** 2021-03-02

**Authors:** Llion Arwyn Roberts, Katsuhiko Suzuki

**Affiliations:** 1 School of Allied Health Sciences, Griffith University, Gold Coast, QLD 4215, Australia; 2 School of Human Movement and Nutrition Sciences, University of Queensland, Brisbane, QLD 4072, Australia; 3 Faculty of Sport Sciences, Waseda University, Tokorozawa, Saitama 359-1192, Japan

Trends relating to specific diets and lifestyle factors like physical (in) activity have formed in recent times. These have increased the calling for research into optimizing their sole and combined effects on health and wellbeing. Maintaining and promoting health and wellbeing involves complex and multifaceted physiological processes, whereby inflammation and oxidative stress are often considered important pillars [1]. Therefore, understanding how these pillars present themselves in isolation and in combination with each other is an important contribution to understanding both the prevention and treatment of diseases. In the context of diet, the scenario is complicated by different nutritional intakes being able to both increase and decrease inflammation and oxidative stress. Congruently, lifestyle factors such as physical activity and inactivity can induce similar responses, with maintaining adequate physical activity levels being attributed to decreasing chronic low-grade inflammation and reducing instances of fatigue and disorders [2]. Therefore, as meeting (or not) appropriate dietary and lifestyle factors can directly influence health and wellbeing, it is imperative to garner further knowledge surrounding their interaction. 

The aim of this issue was to collect and synthesize pertinent literature from original investigations to review articles surrounding these areas, and to provide evidence-based source to enhance our understanding. Collectively, this Special Issue includes 12 empirical investigations that covered multiple aspects of inflammation and oxidative stress from the perspectives of specific nutrients, such as alpha-lipoic acid [3] and sulforaphane [4], and 6 distinct review articles that aimed to consolidate and summarize our understanding of diverse contexts, such as exercising within hypoxia [5], and the influence of diet and dietary antioxidants on mood and depression [6]. In a fundamental review for this issue, Taherkhani and colleagues [7] provide an overview of the mechanistic effects of physical activity on inflammatory cytokines and oxidative stress and how dietary antioxidant consumption can modulate these responses. They highlight the complexity of the state of play, while also drawing attention to the double-edged sword. In this regard, it is important to be mindful of the negative consequences of inflammation and oxidative stress; however, they can play important roles in themselves in triggering adaptive signaling processes after, for example, exercise and/or nutritional intake. In this regard, Li and colleagues [5] provide a complementary insight into relevant molecular mechanisms that govern adaptations to a popular exercise modality, high-intensity interval training, and hypoxia exposure. High-intensity interval training is a popular training modality for healthy individuals but, counterintuitively in some individuals’ minds, is also gaining momentum as a training modality for diseased conditions [8]. In a similarly counterintuitive manner, exposure to hypoxia has also been recognized for its unexpected and counterintuitive benefits, especially on motor function [9]. As such, Li and colleagues’ review [5] offers a valuable overview of how these interventions induce their effects to lay a foundation to explore their combined stimuli to benefit health and disease prevention. 

Three subsequent reviews have focused on the implications of dietary habits, from the specific dietary perspectives of sulforaphane [10] and terpenoids [11], to overall dietary practices [6]. Sulforaphane and terpenoids are classified as nutraceuticals; thus the examination of their potential beneficial effects on health and disease is warranted. From a molecular perspective, sulforaphane is reported to induce strong antioxidant effects to counteract oxidative stress by triggering the antioxidant response element, while concurrently suppressing the proinflammatory regulator, nuclear factor-kappa B. Similarly, terpenoids’ geroprotective functions are summarized on conceptual and molecular bases, including their ability to reduce oxidative stress by stimulating the nuclear factor erythroid-2 to the heme oxygenase-1 pathway, and to induce similar effects of sulforaphane on downregulating the activity of nuclear factor-kappa B and its pathway. Importantly from both reviews, the current state of evidence is summarized and synthesized before providing evidence-based guidelines for future research directions. The final two reviews, the topics of mood and smoking, are covered by Huang and colleagues [6] and Taati and colleagues [12], respectively. While recent data suggest that global initiatives to combat *traditional* smoking are working [13], more information is needed regarding how a popular type of smoking, through a water pipe, influences health outcomes [14]. The review by Taati and colleagues [12] first highlights how similar some of the effects on inflammation and oxidative stress are between water pipe smoking and traditional smoking. However, interesting data are provided regarding not only how water pipe smoking influences aspects of exercise performance but also what ameliorating effects can occur if individuals who partake in water pipe smoking also undertake regular exercise. In a similar vein to water pipe smoking, there is a growing need for data surrounding the links between dietary intake and mood, particularly in some instances where it has long been known that dietary habits can both negatively and positively influence medicine-based outcomes [15]. In Taati and colleagues’ review, they focus on depressive symptoms from the perspective of dietary antioxidants, while also briefly detailing other dietary practices. They highlight not only how education on the benefits of dietary intervention for depression is warranted, but also how beneficial combined therapies, such as diet and exercise, may offer adjuvative effects. 

Seven of the empirical studies in this issue are related specifically to assessing outcomes following dietary consumption in human [16,17,18] and nonhuman [3,4,19,20] models, while three specifically consider the effects of an exercise intervention [21,22,23]. In the human models, interesting results are presented spanning the use of electrolyte-reduced water [16], the dietary correlates of mood across the menstrual cycle [17], and the links between ketogenic diet and microRNA-associated antioxidant status [18]. It was reported that daily consumption of 1.5 liters of electrolyte-reduced water was effective in reducing measures of basal oxidative stress. In other words, a simple diet-based lifestyle change was effective in an occupational setting and, therefore, could be considered a justifiable strategy for reducing oxidative stress-induced impacts on health [16]. While still considering the influences on biological markers of oxidative stress, Cannataro and colleagues [18] demonstrate how consuming a ketogenic diet and microRNAs, particularly miR-30a-5p, are correlated with antioxidant homeostasis, in particular, catalase from utilizing a bioinformatic approach on red blood cell responses. Finally, a cross-sectional study of menstruating females by Bu and colleagues [17] identifies how certain dietary practices correlate with negative mood at different phases of the menstrual cycle (e.g., carbonated beverages during menstruation and fruit intake, e.g., banana and dates in the premenstrual phase). Such information may subsequently be used to tailor dietary strategies and help elucidate future nutraceutical targets for further exploration of their benefits to influence mood across the menstrual cycle. 

In cell and animal models of ischemic–reperfusion injury, selenomethionine was identified as being able to reduce cell necrosis as a consequence of enhanced oxidant scavenging and glutathione peroxidase activity by Reyes and colleagues [20]. However, when translating the results to an in vivo dietary model, supplementation did not overtly improve cardiac tissue function. In a comparative examination involving murine cardiac tissue by Skrzep-Poloczek and colleagues [19], it was interestingly observed that oxidative stress markers (e.g., glutathione reductase) were related to and influenced more by high-fat dietary practices than when a model of bariatric surgery was applied following such a diet. As such, further investigation is required to explore the effects of other dietary patterns on cardiac tissue and oxidative stress to be able to better elucidate effective long-term treatment options. Two specific dietary nutraceuticals were examined in the form of sulforaphane by Ruhee and colleagues [4] and alpha-lipoic acid (LA) by Andreeva-Gateva and colleagues [3]. Ruhee [4] investigated the efficacy of sulforaphane at the prominent site of oxidative stress, the macrophage level. When lipopolysaccharide was used to stimulate inflammation, cells previously exposed to sulforaphane displayed attenuated oxidative stress and inflammation by reductions in nitric oxide and cytokine expression. Similar antioxidant effects were observed following LA administration by Andreeva-Gateva [3]. Interestingly, female mice displayed elevated blood uric acid levels compared with males, which was associated with improved emphysema and lung dysfunction symptoms. As such, strategies to improve oxidative stress may be favorable to counteract some of the symptoms of Chronic Obstructive Pulmonary Disease (COPD). Finally, intestinal epithelial cells were used by Carullo and associates [24] to investigate the effects of a newly developed fortified fermented milk, kefir. Kefir was fortified with pomace seed extract and resultingly displayed enhanced antioxidant properties compared with unfortified kefir. However, neither fortified nor unfortified kefir (control) influenced markers of cell integrity, thus requiring further research into the effectiveness of this particular type of fortified kefir to aid with intestinal inflammation.

When considering the physical activity aspect of an individual’s lifestyle, three investigations have examined responses after endurance-type exercise here, spanning acute single bouts (i.e., a 3 km running time trial [23] and a maximal running test [22]) to 16 weeks of regular cycling [21]. In a detailed multitarget urinary investigation, Tominaga and colleagues [23] identified multiple markers that changed in response to a time trial, which collectively helped identify potential noninvasive parameters that can be used to monitor responses to exercise. Interestingly, only 8-OHdG changed from the perspective of oxidative stress, by decreasing after exercise; however, multiple inflammatory-based markers (e.g., interleukins) changed. In a comparatively noninvasive manner, saliva was examined after maximal running by Arazi and colleagues [22]. However, the key comparison in the investigation was how exercise-induced responses differed between individuals who partook in water pipe smoking and those who did not. Smoking attenuated antioxidant response post-exercise. Therefore, while exercise has previously been attributed to combating some of the negative consequences of smoking chronically, acute responses may require further examination to assist in inducing optimal oxidative stress responses. Lastly, an examination of the effects of endurance training in the elderly was undertaken from the perspective of the links between pentraxin 3 and oxidative stress. While markers of oxidative stress remained unchanged in the intervention, pentraxin was reduced. As such, while links between pentraxin 3 and oxidative stress were proposed, the association was not apparent in the elderly. However, the study provided insights into other anti-inflammatory mechanisms that involve pentraxin 3 that require future investigation.

Collectively, while there is a growing belief in the benefits of exercise as a lifestyle strategy to enhance overall wellbeing, it is important to consider the contribution that diet can make. This Special Issue has identified many examples of where diet or exercise or a combination of the two can beneficially influence oxidative stress and inflammation, or highlights particular elements that require future research to optimize approaches. Using diet specifically to help influence nonlifestyle disorders is currently uncommon. However, the ability of nutraceuticals to positively reduce important contributory factors and mechanisms of disease requires detailed future research to elucidate their ability to be of benefit to consumers independently or concurrently [25].

## References

[1] Roberts L., Suzuki K. (2019). Exercise and Inflammation. Antioxidants.

[2] Suzuki K. (2019). Chronic Inflammation as an Immunological Abnormality and Effectiveness of Exercise. Biomolecules.

[3] Andreeva-Gateva P., Traikov L., Sabit Z., Bakalov D., Tafradjiiska-Hadjiolova R. (2020). Antioxidant Effect of Alpha-Lipoic Acid in 6-Hydroxydopamine Unilateral Intrastriatal Injected Rats. Antioxidants.

[4] Ruhee R.T., Ma S., Suzuki K. (2019). Sulforaphane Protects Cells against Lipopolysaccharide-Stimulated Inflammation in Murine Macrophages. Antioxidants.

[5] Li J., Li Y., Atakan M.M., Kuang J., Hu Y., Bishop D.J., Yan X. (2020). The Molecular Adaptive Responses of Skeletal Muscle to High-Intensity Exercise/Training and Hypoxia. Antioxidants.

[6] Huang Q., Liu H., Suzuki K., Ma S., Liu C. (2019). Linking What We Eat to Our Mood: A Review of Diet, Dietary Antioxidants, and Depression. Antioxidants.

[7] Taherkhani S., Suzuki K., Castell L. (2020). A Short Overview of Changes in Inflammatory Cytokines and Oxidative Stress in Response to Physical Activity and Antioxidant Supplementation. Antioxidants.

[8] Gibala M.J., Little J.P., Macdonald M.J., Hawley J.A. (2012). Physiological adaptations to low-volume, high-intensity interval training in health and disease. J. Physiol..

[9] Dale E.A., Ben Mabrouk F., Mitchell G.S. (2014). Unexpected benefits of intermittent hypoxia: Enhanced respiratory and nonrespiratory motor function. Physiology (Bethesda).

[10] Ruhee R.T., Suzuki K. (2020). The Integrative Role of Sulforaphane in Preventing Inflammation, Oxidative Stress and Fatigue: A Review of a Potential Protective Phytochemical. Antioxidants.

[11] Proshkina E., Plyusnin S., Babak T., Lashmanova E., Maganova F., Koval L., Platonova E., Shaposhnikov M., Moskalev A. (2020). Terpenoids as Potential Geroprotectors. Antioxidants.

[12] Taati B., Arazi H., Suzuki K. (2020). Oxidative Stress and Inflammation Induced by Waterpipe Tobacco Smoking Despite Possible Protective Effects of Exercise Training: A Review of the Literature. Antioxidants.

[13] (2019). WHO Global Report on Trends in Prevelance of Tobacco Use 2000–2025.

[14] Aboaziza E., Eissenberg T. (2015). Waterpipe tobacco smoking: What is the evidence that it supports nicotine/tobacco dependence?. Tobacco Control.

[15] Rao T.S.S., Asha M.R., Ramesh B.N., Rao K.S.J. (2008). Understanding nutrition, depression and mental illnesses. Indian J. Psychiatry.

[16] Choi Y.A., Lee D.H., Cho D.-Y., Lee Y.-J. (2020). Outcomes Assessment of Sustainable and Innovatively Simple Lifestyle Modification at the Workplace-Drinking Electrolyzed-Reduced Water (OASIS-ERW): A Randomized, Double-Blind, Placebo-Controlled Trial. Antioxidants.

[17] Bu L., Lai Y., Deng Y., Xiong C., Li F., Li L., Suzuki K., Ma S., Liu C. (2020). Negative Mood Is Associated with Diet and Dietary Antioxidants in University Students During the Menstrual Cycle: A Cross-Sectional Study from Guangzhou, China. Antioxidants.

[18] Cannataro R., Caroleo M.C., Fazio A., La Torre C., Plastina P., Gallelli L., Lauria G., Cione E. (2019). Ketogenic Diet and microRNAs Linked to Antioxidant Biochemical Homeostasis. Antioxidants.

[19] Skrzep-Poloczek B., Poloczek J., Chełmecka E., Dulska A., Romuk E., Idzik M., Kazura W., Nabrdalik K., Gumprecht J., Jochem J. (2020). The Oxidative Stress Markers in the Erythrocytes and Heart Muscle of Obese Rats: Relate to a High-Fat Diet but Not to DJOS Bariatric Surgery. Antioxidants.

[20] Reyes L., Bishop D.P., Hawkins C.L., Rayner B.S. (2019). Assessing the Efficacy of Dietary Selenomethionine Supplementation in the Setting of Cardiac Ischemia/Reperfusion Injury. Antioxidants.

[21] Estébanez B., Rodriguez A.L., Visavadiya N.P., Whitehurst M., Cuevas M.J., González-Gallego J., Huang C.-J. (2020). Aerobic Training Down-Regulates Pentraxin 3 and Pentraxin 3/Toll-Like Receptor 4 Ratio, Irrespective of Oxidative Stress Response, in Elderly Subjects. Antioxidants.

[22] Arazi H., Taati B., Rafati Sajedi F., Suzuki K. (2019). Salivary Antioxidants Status Following Progressive Aerobic Exercise: What Are the Differences between Waterpipe Smokers and Non-Smokers?. Antioxidants.

[23] Tominaga T., Ma S., Sugama K., Kanda K., Omae C., Choi W., Hashimoto S., Aoyama K., Yoshikai Y., Suzuki K. (2021). Changes in Urinary Biomarkers of Organ Damage, Inflammation, Oxidative Stress, and Bone Turnover Following a 3000-m Time Trial. Antioxidants.

[24] Carullo G., Governa P., Spizzirri U.G., Biagi M., Sciubba F., Giorgi G., Loizzo M.R., Di Cocco M.E., Aiello F., Restuccia D. (2020). Sangiovese cv Pomace Seeds Extract-Fortified Kefir Exerts Anti-Inflammatory Activity in an In Vitro Model of Intestinal Epithelium Using Caco-2 Cells. Antioxidants.

[25] Suzuki K., Tominaga T., Ruhee R.T., Ma S. (2020). Characterization and Modulation of Systemic Inflammatory Response to Exhaustive Exercise in Relation to Oxidative Stress. Antioxidants.

